# Regulation of Cell Viability and Anti-inflammatory Tristetraprolin Family Gene Expression in Mouse Macrophages by Cottonseed Extracts

**DOI:** 10.1038/s41598-020-57584-9

**Published:** 2020-01-21

**Authors:** Heping Cao, Kandan Sethumadhavan

**Affiliations:** United States Department of Agriculture, Agricultural Research Service, Southern Regional Research Center, 1100 Robert E. Lee Boulevard, New Orleans, LA 70124 USA

**Keywords:** RNA, Acute inflammatory arthritis

## Abstract

Bioactive plant extracts have been used for the prevention and treatment of various diseases. One of the major classes of bioactive compounds is plant polyphenols. Cottonseed ethanol extracts were determined by HPLC-MS analysis to be essentially free of toxic gossypol. The objective of this study was to investigate the effect of cottonseed ethanol extracts on the cytotoxicity and regulation of anti-inflammatory tristrataprolin (TTP) family gene expression in mouse cells. MTT, qPCR and immunoblotting assays tested the effects of cottonseed extracts in mouse RAW264.7 macrophages and 3T3-L1 adipocytes. No cytotoxicity effect was observed in macrophages treated with extracts from the coat or kernel of glanded and glandless cottonseed. Similarly, the viability of mouse adipocytes was not affected by cottonseed extracts. In contrast, gossypol and lipopolysaccharides were toxic to macrophages but not adipocytes under high concentration or long time treatment. Cottonseed extracts exhibited modest effect on TTP family gene expression in macrophages but glandless cottonseed coat extract significantly increased TTP mRNA and protein levels with a magnitude similar to cinnamon and green tea polyphenol extract and insulin. These results demonstrated that cottonseed extracts are harmless towards the mouse cells and that glandless cottonseed coat extract stimulates TTP gene expression. We propose that glandless cottonseed is a safe source of plant polyphenols with anti-inflammatory property.

## Introduction

*Gossypium hirsutum* L. (Cotton) produces fiber and cottonseed, two economically important commodities. Cottonseed weights more than fiber but values for 20% of the crop^1^. Cottonseed is classified as glanded or glandless according to the presence or absence of pigmented gossypol glands^2,3^. Glanded cottonseed contains bioactive compounds including gossypol^4^, gallic acid^5^, 3,4-dihydroxybenzoic acid^5^, bioactive peptides^6^, and flavonol glycosides^5^. Glandless cottonseed also contains many bioactive chemicals including the antidepressant compound quercetin^7^. These bioactive components could be targeted for increasing the value of cottonseed with health promotion and disease prevention potentials.

Plant bioactive products have long been used for disease prevention and treatment. Plant polyphenols are major bioactive compounds accumulated in various plant tissues. These polyphenol compounds are generated from the plant flavonoid biosynthetic pathway and used for plant defenses against predators^8^. Plant polyphenols are discovered to be present in most diet and beneficial to human health^9,10^.

Plant polyphenols are shown to regulate gene expression in numerous studies. For example, green tea polyphenols regulate the expression of many genes in rats under a high fructose diet feeding^11,12^. Cinnamon polyphenols regulate the expression of genes coding for proteins in the insulin signaling pathway, inflammatory responses and lipid metabolism^13–17^. Plant polyphenols are generally water-soluble and extracted by ethanol from cinnamon tree barks and by hot water from green tea leaves. In contrast, toxic compounds such as cinnamaldehyde (essential oil) are extracted by organic solvents^12,15,18,19^. We recently developed protocols for isolating bioactive ethanol extracts which were shown by HPLC-MS to be essentially free of gossypol from glanded and glandless cottonseed^20^. These bioactive cottonseed extracts affect human cancer cell growth and mouse gene expression coding for diacylglycerol acyltransferase (DGAT) and human antigen R (HuR)^20–22^.

Tristetraprolin/zinc finger protein 36 (TTP/ZFP36) and its homologues are anti-inflammatory proteins^23,24^. TTP family consists of four homologues in mice and rats (ZFP36/TTP, ZFP36L1/TIS11B, ZFP36L2/TIS11D, andZFP36L3)^25,26^. TTP binds to some cytokine mRNA AU-rich elements and destabilizes those molecules^27,28^. TTP knockout mice accumulate excessive levels of the proinflammatory cytokines and develop a systemic inflammatory syndrome consisting of arthritis, autoimmunity, and myeloid hyperplasia^29,30^. TTP over-expression decreases inflammation in macrophages^31^. Therefore, chemicals that increase TTP expression may have therapeutic value for inflammation-related disease prevention and/or treatment. However, it is not known whether cottonseed components can regulate TTP family gene expression since no prior work was done in this area.

The aim of current study was to investigate the effects of cottonseed extracts on the viability and regulation of TTP family gene expression in mouse cells. We used MTT, qPCR and immunoblotting assays to investigate cottonseed extract effects on mouse cell viability and the expression of anti-inflammatory TTP family genes^32,33^. Our results showed that cottonseed extracts are harmless towards the mouse cells and that glandless cottonseed coat extract stimulates TTP gene expression. We propose that glandless cottonseed is a safe source of plant polyphenols with anti-inflammatory property.

## Results

### Effect of cottonseed extracts on macrophage viability

**Figure 1:**
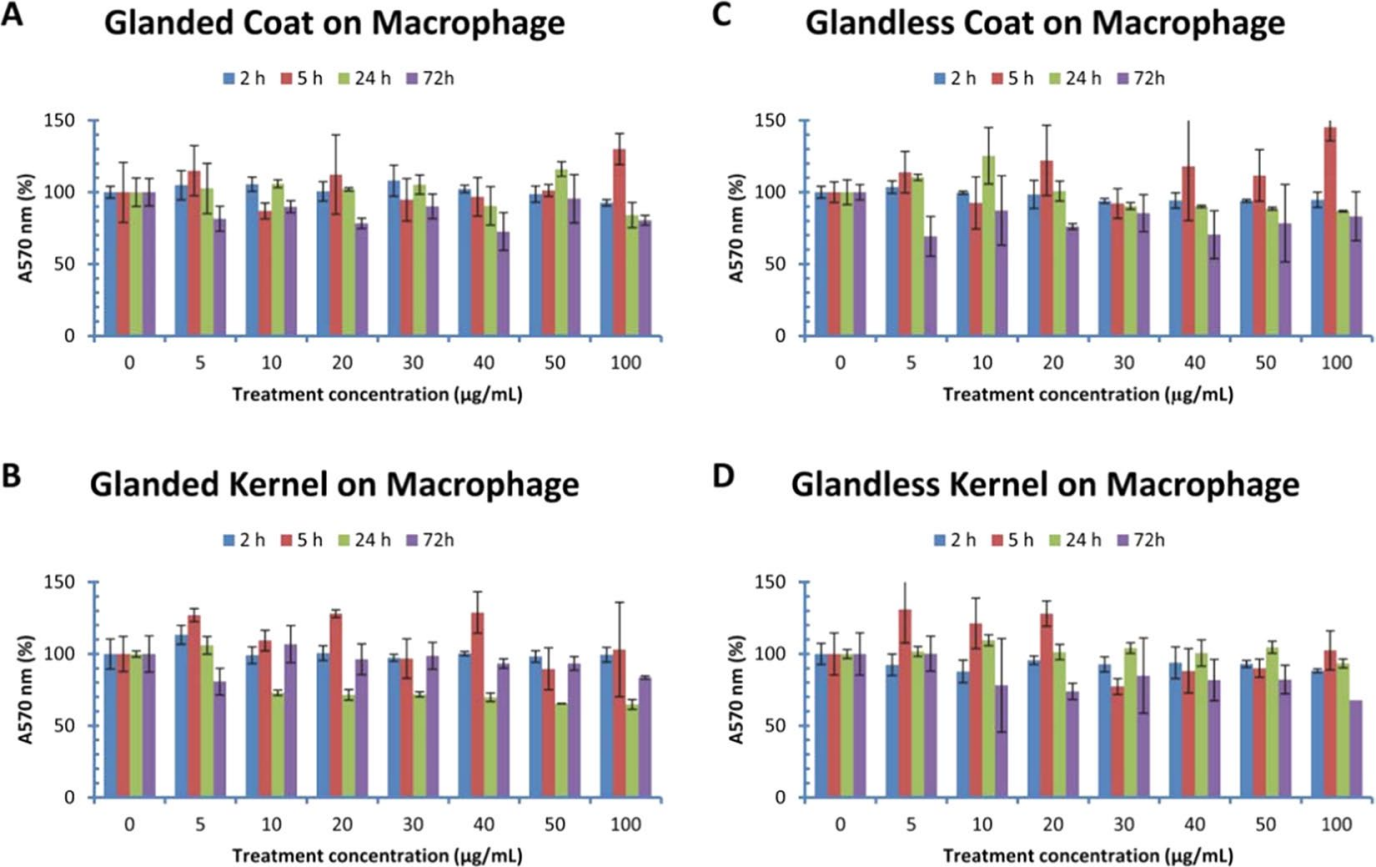
Effect of cottonseed extracts on mouse macrophage viability. RAW264.7 macrophages were treated with cottonseed extracts for 2, 5, 24 and 72 h. (**A**) glanded cottonseed coat extract, (**B**) glanded cottonseed kernel extract, (**C**) glandless cottonseed coat extract, (**D**) glandless cottonseed kernel extract. The data represent the mean and standard deviation of three independent samples.

MTT method was used to determine cell viability after being treated with cottonseed extracts for 2–72 h (Fig. 1). The viability of macrophages was not statistically affected by glanded cottonseed coat extract (Fig. 1A). Glanded cottonseed kernel extract also did not show significant effect on macrophage viability (Fig. 1B). Similar experiments were conducted on RAW cell viability using extracts from glandless cottonseed. MTT assays showed that extracts from coat (Fig. 1C) and kernel (Fig. 1D**)** of glandless cottonseed did not have significant effect on RAW cell viability after 2–72 h treatment with 5–100 µg/mL of the extracts. However, macrophage viability appeared to be reduced slightly, although not significantly, by higher concentration and longer time of the cottonseed extract treatment (Fig. 1A–D).

### Effect of cottonseed extracts on adipocyte viability

**Figure 2:**
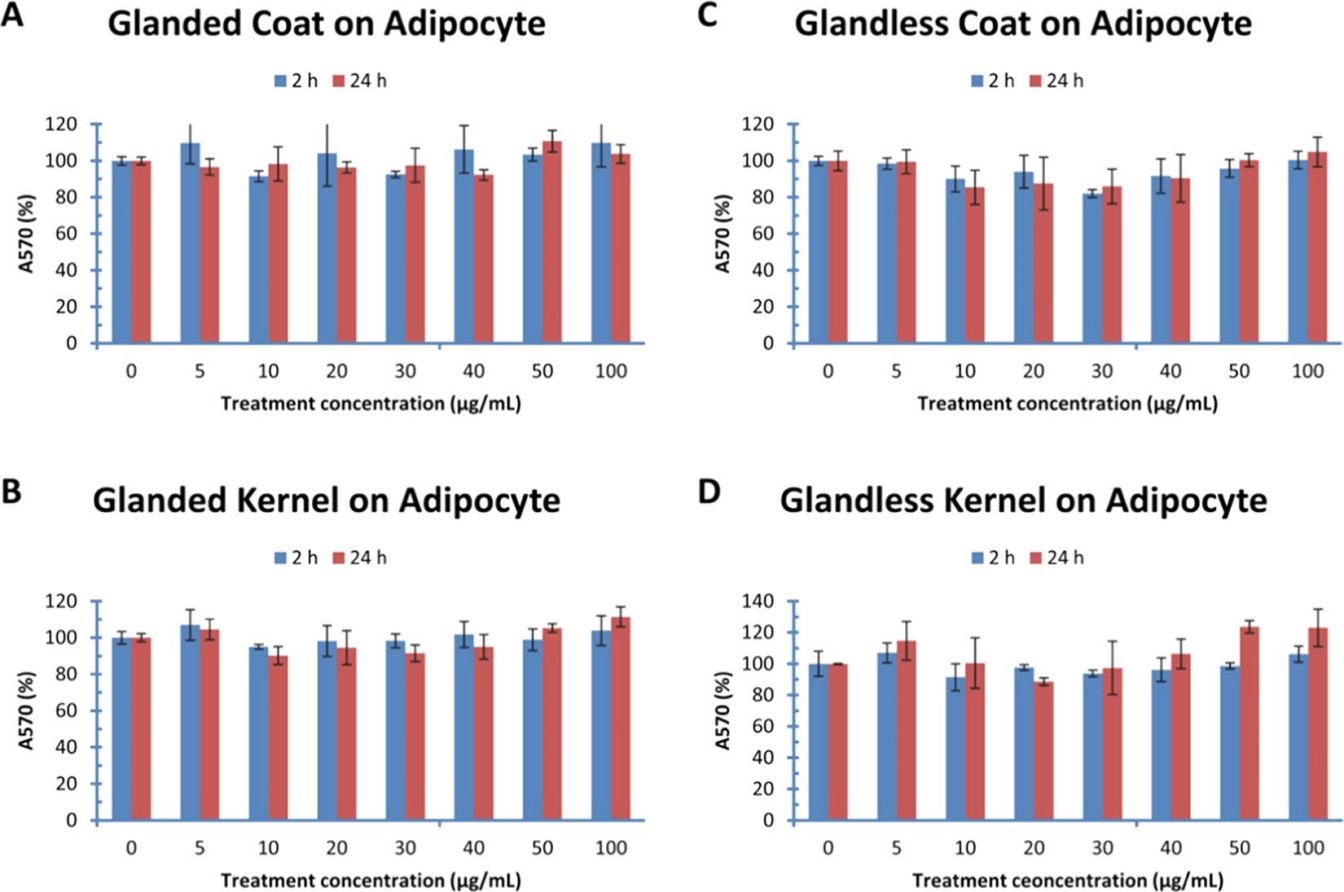
Effect of cottonseed extracts on mouse adipocyte viability. Mouse 3T3-L1 adipocytes were treated with cottonseed extracts for 2 and 24 h. (**A**) glanded cottonseed coat extract, (**B**) glanded cottonseed kernel extract, (**C**) glandless cottonseed coat extract, (**D**) glandless cottonseed kernel extract. The data represent the mean and standard deviation of three independent samples.

The viability of mouse adipocytes was also determined with MTT method after being treated with cottonseed extracts for 2–24 h (Fig. 2). The adipocyte viability was not affected statistically by any of the treatments using glanded cottonseed coat or kernel extract up to 100 µg/mL for 2 or 24 h (Fig. 2A,B). Similarly, extracts from glandless cottonseed coat (Fig. 2C) and kernel (Fig. 2D**)** did not have significant effect on adipocyte viability after treatment for 2–24 h with 5–100 µg/mL of the extracts.

### Effect of gossypol and lipopolysaccharides on cell cytotoxicity

**Figure 3:**
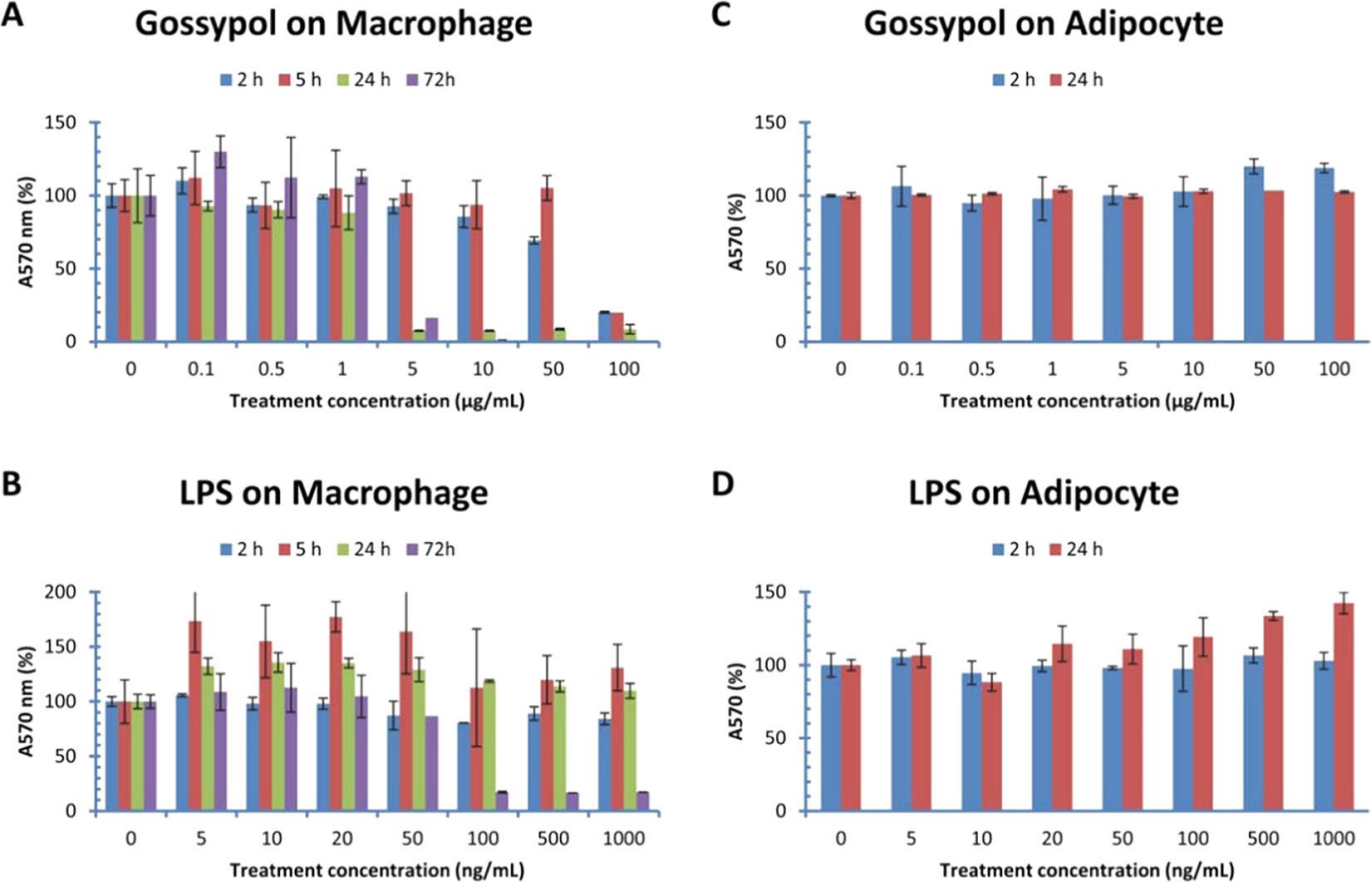
Effect of gossypol and LPS on mouse cell viability. Mouse RAW264.7 macrophages were treated with gossypol and LPS for 2, 5, 24 and 72 h, and 3T3-L1 adipocytes treated for 2 and 24 h. (**A**) gossypol effect in macrophages, (**B**) LPS effect in macrophages, (**C**) gossypol effect in adipocytes, (**D**) LPS in adipocytes. The data represent the mean and standard deviation of three independent samples.

As controls for the experiments, cell viability was measured in macrophages and adipocytes treated with the cytotoxic compound gossypol (known to be accumulated in the glanded cottonseed which causes male infertility) and the well-known endotoxin lipopolysaccharides (LPS). MTT assays showed that gossypol exhibited significant inhibitory effect on RAW macrophage viability under high concentration or long treatment time (Fig. 3A). Mitochondrial activity in RAW macrophages was almost completely inhibited by gossypol after treatment at 5–50 µg/mL for 24–72 h or 100 µg/mL for 2–72 h (Fig. 3A). MTT assays also showed that LPS had significant inhibitory effect on RAW macrophage survival after 100–1000 ng/mL treatment for 72 h (Fig. 3B). However, MTT assays showed that gossypol and LPS did not have significant effect on adipocyte survival after 2–24 h treatments (Fig. 3C,D).

### Effect of cottonseed extracts on TTP/ZFP36 gene expression

**Figure 4:**
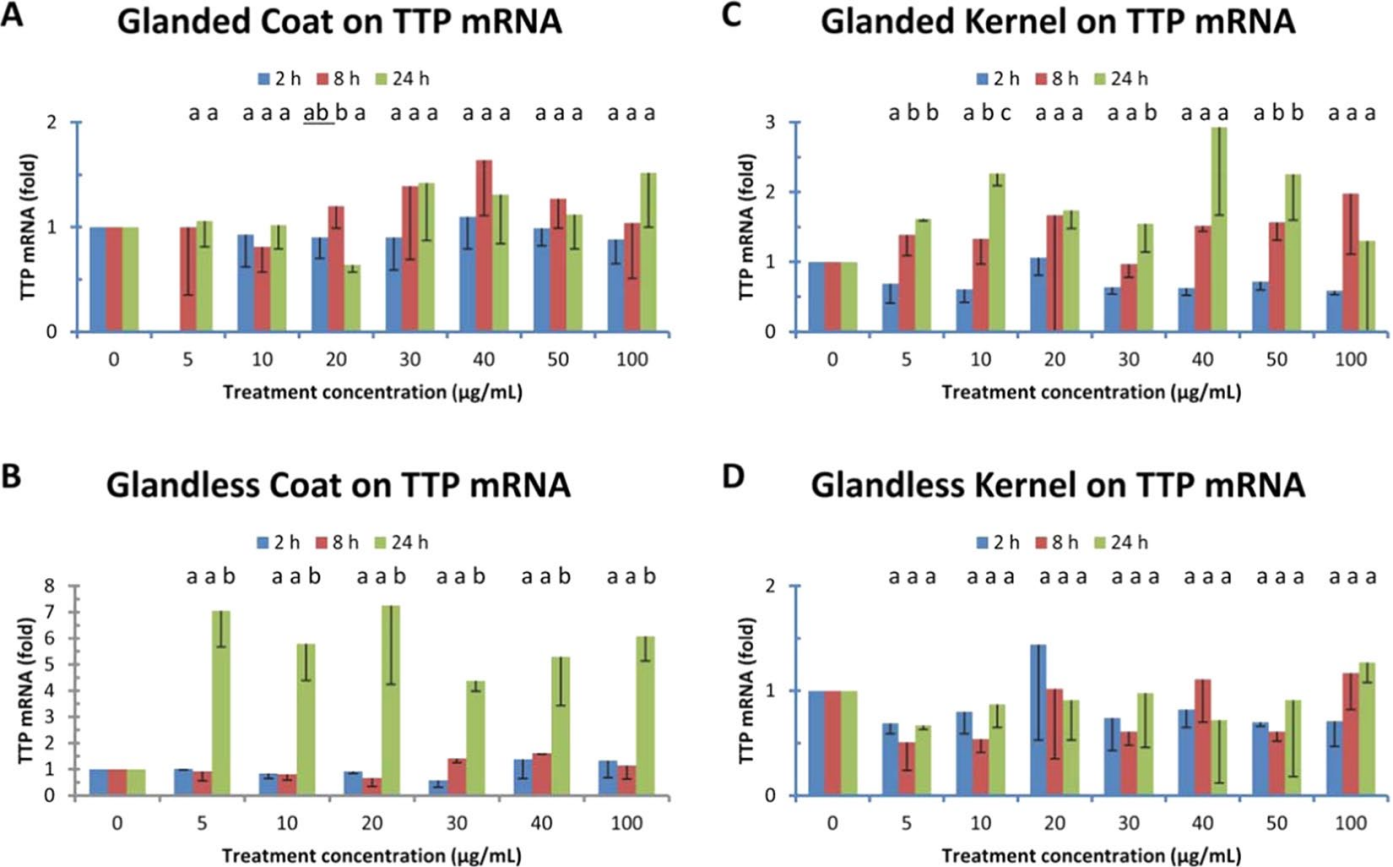
Effect of cottonseed extracts on TTP mRNA expression. Mouse RAW264.7 macrophages were treated with the extract (0–100 µg/mL, “0” treatment corresponding to 1% DMSO in the culture medium) for 2, 8 and 24 h. Total RNAs were isolated from the cells and used for cDNA synthesis. The SYBR Green qPCR reaction mixtures contained 5 ng of RNA-equivalent cDNAs from each sample and 200 nM of each primer. The 2^−ΔΔ*CT*^ method of relative quantification was used to determine the fold change in expression using RPL32 mRNA as a reference mRNA. The data represent the mean and standard deviation of three independent samples. Different lower case letters displayed above each of the treatment time on the figures are significantly different between the LPS concentrations at p < 0.05. (**A**) glanded cottonseed coat extract, (**B**) glanded cottonseed kernel extract, (**C**) glandless cottonseed coat extract, (**D**) glandless cottonseed kernel extract.

The above results suggest that cottonseed extracts were not toxic to mouse macrophages and adipocytes under the experimental conditions. To provide evidence for potential health and nutritional benefits of this abundant resource of plant polyphenols, the effect of cottonseed extracts on anti-inflammatory TTP gene expression was investigated using mouse macrophages. qPCR showed that the effect of glanded cottonseed coat extract on TTP mRNA levels was minimal to modest in mouse RAW macrophages (Fig. 4A). TTP mRNA levels were increased less than two-fold without statistical significance in macrophages treated for 2, 8 and 24 h. Similarly, the kernel extract from glanded cottonseed showed less than three-fold of increases in TTP mRNA levels after 24 h treatment (Fig. 4B). The coat extract from glandless cottonseed did not have significant effects on TTP mRNA levels after 2–8 h treatment. However, TTP mRNA levels were significantly increased up to 7-fold in 24 h treated-macrophages by glandless coat extract (Fig. 4C). In contrast, the effect of kernel extract from glandless cottonseed on TTP mRNA levels was minimal with no statistical significance in the mouse RAW macrophages (Fig. 4D).

**Figure 5:**
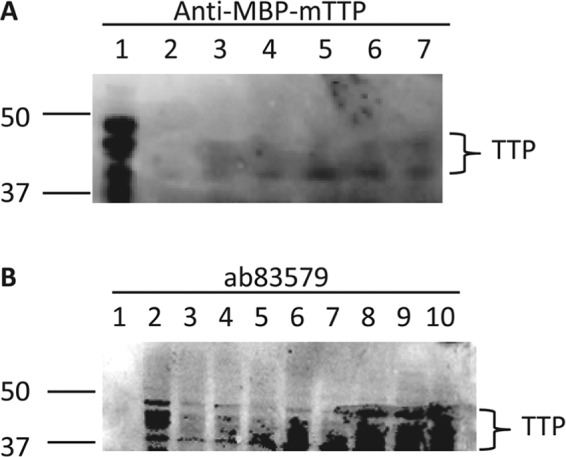
Effect of LPS and cottonseed extracts on TTP protein expression. Mouse RAW264.7 cells were stimulated with LPS and cottonseed extracts for various times. Cell extract was used for immunoblotting with the anti-MBP-mTTP serum or synthetic peptide ab83579 polyclonal antibodies. The blot was incubated in the primary antibodies for 18 h and the secondary antibody for 4 h. (**A**) anti-MBP-mTTP serum, lane 1: LPS (2 h, 100 ng/mL), lanes 2–7: glandless cottonseed coat extract (24 h, 5, 10, 20, 30, 40 and 100 µg/mL, respectively). (**B**) ab83579, lane 1: protein standards, lane 2: LPS (2 h, 100 ng/mL), lanes 3–6: glanded cottonseed coat extract (100 µg/mL, 2, 4, 8 and 24 h, respectively), lanes 7–10: glandless cottonseed coat extract (100 µg/mL, 2, 4, 8 and 24 h, respectively). The full immunoblot is presented as a Supplementary Figure.

Immunoblotting was used to confirm if increased TTP mRNA levels by glandless cottonseed coat extract could result in increased TTP protein levels (Fig. 5). TTP polyclonal antibodies were used to detect TTP protein in macrophages treated with cottonseed extracts using LPS as a positive control, a well-known agent to induce TTP protein expression and phosphorylation in mouse macrophages^14,34^. Immune-reactive band(s) between 37 and 50 kDa corresponding to the predicted sizes of TTP and its phosphorylated forms were detected in the cells treated for 2 h with 100 ng/mL of LPS (Fig. 5A, lane 1 and Fig. 5B, lane 2). Similar sizes of immune-reactive bands with less intensity were detected by anti-MBP-mTTP antibodies in macrophages treated for 24 h with 5, 10, 20, 30, 40 and 100 µg/mL of glandless cottonseed coat extract (Fig. 5A). Similar immune-reactive bands were detected by synthetic TTP peptide antibodies in macrophages treated with 100 µg/mL of glandless cottonseed coat extract (lanes 7–10), and their levels were more than those treated with glanded cottonseed coat extract (lanes 3–6) (Fig. 5B).

### Effect of cottonseed extracts on ZFP36L1 gene expression

Analyses of the effects of cottonseed extracts on gene expression were extended to the other three homologues of TTP in mouse macrophages. The effect of coat or kernel extracts from glanded or glandless cottonseed on ZFP36L1 gene mRNA levels in mouse RAW macrophages was minimal, mostly without statistical significance (Table 1). ZFP36L1 mRNA levels were increased approximately two-fold in macrophages treated for 2 h with most of the glanded coat extract concentrations but the effects were reduced to minimal or even less than the control after 8–24 h treatment (Table 1). The effect of kernel extract from glanded cottonseed on ZFP36L1 mRNA levels showed less than the control after 2–8 h treatment and returned to normal in 24 h treatment (Table 1). The effect of coat extract from glandless cottonseed on ZFP36L1 mRNA levels in mouse RAW macrophages was also minimal except an increase of ZFP36L1 mRNA levels in 30 µg/mL treated macrophages (Table 1). The effect of kernel extract from glandless cottonseed on ZFP36L1 mRNA levels showed similar pattern of glanded cottonseed kernel extract with less than the control after 2–8 h treatment and returned to normal in 24 h treatment (Table 1).

**Table 1:** Effect of Cottonseed Extracts on ZFP36L1 mRNA Levels.

Glanded coat	2 h	8 h	24 h
1% DMSO	1.00 ± 0.00a	1.00 ± 0.00a	1.00 ± 0.00a
5 µg/ml	2.05 ± 1.20a	0.68 ± 0.32a	0.77 ± 0.18a
10 µg/ml	1.36 ± 0.36a	0.49 ± 0.05b	0.86 ± 0.25ab
20 µg/ml	0.99 ± 0.24a	0.58 ± 0.09a	0.72 ± 0.18a
30 µg/ml	1.50 ± 0.91a	0.62 ± 0.21a	1.00 ± 0.37a
40 µg/ml	2.38 ± 0.68a	0.63 ± 0.35b	1.00 ± 0.19b
50 µg/ml	1.27 ± 0.29a	0.79 ± 0.30a	1.02 ± 0.11a
100 µg/ml	2.45 ± 0.45a	0.43 ± 0.22b	1.08 ± 0.32b
**Glanded kernel**	**2 h**	**8 h**	**24 h**
1% DMSO	1.00 ± 0.00a	1.00 ± 0.00a	1.00 ± 0.00a
5 µg/ml	0.50 ± 0.12a	1.16 ± 0.37a	1.52 ± 0.33b
10 µg/ml	0.43 ± 0.17a	0.67 ± 0.27a	1.00 ± 0.28a
20 µg/ml	0.68 ± 0.17a	0.83 ± 0.79a	1.41 ± 0.43a
30 µg/ml	0.55 ± 0.38a	0.76 ± 0.08a	1.14 ± 0.39a
40 µg/ml	0.51 ± 0.28a	0.80 ± 0.15a	1.07 ± 0.38a
50 µg/ml	0.39 ± 0.24a	1.61 ± 0.30b	1.36 ± 0.36b
100 µg/ml	0.31 ± 0.22a	0.90 ± 0.11b	0.92 ± 0.21b
**Glandless coat**	**2 h**	**8 h**	**24 h**
1% DMSO	1.00 ± 0.00a	1.00 ± 0.00a	1.00 ± 0.00a
5 µg/ml	1.14 ± 0.24a	0.91 ± 0.40a	1.19 ± 0.36a
10 µg/ml	0.71 ± 0.14a	0.76 ± 0.25a	1.08 ± 0.27a
20 µg/ml	1.36 ± 0.90a	0.76 ± 0.42a	0.98 ± 0.25a
30 µg/ml	1.56 ± 1.10a	2.02 ± 0.79a	1.27 ± 0.38a
40 µg/ml	1.15 ± 0.48a	1.00 ± 0.45a	1.07 ± 0.02a
50 µg/ml	0.89 ± 0.00a	1.03 ± 0.10a	1.05 ± 0.05a
100 µg/ml	0.92 ± 0.04a	0.68 ± 0.63a	0.90 ± 0.13a
**Glandless kernel**	**2 h**	**8 h**	**24 h**
1% DMSO	1.00 ± 0.00a	1.00 ± 0.00a	1.00 ± 0.00a
5 µg/ml	0.60 ± 0.15a	0.52 ± 0.06a	0.81 ± 0.20a
10 µg/ml	0.52 ± 0.10a	0.66 ± 0.18a	1.10 ± 0.28b
20 µg/ml	1.06 ± 0.66a	0.62 ± 0.26a	1.35 ± 0.21a
30 µg/ml	0.37 ± 0.22a	0.48 ± 0.07a	1.00 ± 0.14b
40 µg/ml	0.37 ± 0.08a	0.85 ± 0.29a	0.84 ± 0.84a
50 µg/ml	0.34 ± 0.11a	0.38 ± 0.02a	1.57 ± 0.77b
100 µg/ml	0.41 ± 0.11a	0.56 ± 0.34a	0.72 ± 0.74a

The data represent the mean and standard deviation of three independent samples. Different lower case letters displayed on the right side of the data in each column are significantly different between the cottonseed extract concentrations at p < 0.05. Values with statistical significance in gene expression are underlined.

### Effect of cottonseed extracts on ZFP36L2 gene expression

ZFP36L2 mRNA levels in mouse RAW macrophages were minimally to modestly affected by the extracts from coat or kernel extracts from glanded or glandless cottonseed (Table 2). ZFP36L2 mRNA levels were not affected by the coat extract from glanded cottonseed in mouse RAW macrophages treated for 2, 8 or 24 h except at 100 µg/ml treatment (Table 2). However, ZFP36L2 mRNA levels in macrophages were significantly increased by glanded kernel extract up to 3-fold (20 µg/mL for 8 h) and 4-fold (10 µg/mL for 24 h) (Table 2). The coat extract from glandless cottonseed increased ZFP36L2 mRNA levels less than two-fold but with statistical significance in 24 h treated macrophages (Table 2). The effect of kernel extract from glandless cottonseed on ZFP36L2 mRNA levels in mouse RAW macrophages was similar to glanded cottonseed kernel extract with less than the control in 2–8 h treated cells and less effective than the kernel extract of glanded cottonseed in 24 h treated cells (Table 2).

**Table 2:** Effect of Cottonseed Extracts on ZFP36L2 mRNA Levels.

Glanded coat	2 h	8 h	24 h
1% DMSO	1.00 ± 0.00a	1.00 ± 0.00a	1.00 ± 0.00a
5 µg/ml	0.89 ± 0.28a	0.96 ± 0.57a	0.86 ± 0.22a
10 µg/ml	0.99 ± 0.44a	0.77 ± 0.24a	1.13 ± 0.07a
20 µg/ml	0.59 ± 0.05a	0.76 ± 0.22a	0.58 ± 0.21a
30 µg/ml	0.96 ± 0.66a	0.78 ± 0.27a	1.32 ± 0.42a
40 µg/ml	1.27 ± 0.91a	1.26 ± 0.38a	1.15 ± 0.20a
50 µg/ml	0.56 ± 0.26a	1.17 ± 0.20a	0.83 ± 0.30a
100 µg/ml	2.06 ± 0.69a	0.67 ± 0.18b	1.07 ± 0.30b
**Glanded kernel**	**2 h**	**8 h**	**24 h**
1% DMSO	1.00 ± 0.00a	1.00 ± 0.00a	1.00 ± 0.00a
5 µg/ml	0.66 ± 0.09a	1.51 ± 0.02b	2.76 ± 0.50 c
10 µg/ml	0.75 ± 0.47a	1.28 ± 0.89a	3.77 ± 1.08b
20 µg/ml	1.03 ± 0.26a	3.05 ± 0.60b	2.96 ± 0.41b
30 µg/ml	0.65 ± 0.25a	0.92 ± 0.33a	1.46 ± 0.40a
40 µg/ml	0.54 ± 0.22a	1.37 ± 0.20b	1.92 ± 0.27 c
50 µg/ml	0.46 ± 0.13a	1.11 ± 0.51a	2.06 ± 1.20a
100 µg/ml	0.43 ± 0.08a	1.63 ± 0.39a	1.52 ± 0.94a
**Glandless coat**	**2 h**	**8 h**	**24 h**
1% DMSO	1.00 ± 0.00a	1.00 ± 0.00a	1.00 ± 0.00a
5 µg/ml	1.16 ± 0.25a	1.57 ± 0.76a	1.51 ± 0.17a
10 µg/ml	0.58 ± 0.26a	1.65 ± 0.19b	1.44 ± 0.07b
20 µg/ml	0.85 ± 0.77a	1.52 ± 0.92a	0.95 ± 0.08a
30 µg/ml	0.97 ± 0.18a	2.62 ± 0.23b	1.48 ± 0.38 c
40 µg/ml	1.11 ± 0.40a	1.74 ± 1.13a	1.15 ± 0.01a
50 µg/ml	1.22 ± 0.49a	1.64 ± 0.35a	1.12 ± 0.09a
100 µg/ml	0.82 ± 0.27a	2.01 ± 0.34b	1.06 ± 0.07a
**Glandless kernel**	**2 h**	**8 h**	**24 h**
1% DMSO	1.00 ± 0.00a	1.00 ± 0.00a	1.00 ± 0.00a
5 µg/ml	0.70 ± 0.21a	0.50 ± 0.11a	0.70 ± 0.16a
10 µg/ml	0.72 ± 0.07a	0.45 ± 0.13b	1.11 ± 0.17 c
20 µg/ml	1.11 ± 0.72a	0.49 ± 0.23a	1.71 ± 1.17a
30 µg/ml	0.62 ± 0.36a	0.68 ± 0.15a	1.23 ± 0.19a
40 µg/ml	0.42 ± 0.08a	0.63 ± 0.41a	0.76 ± 0.55a
50 µg/ml	0.36 ± 0.12a	0.57 ± 0.12a	1.34 ± 1.04a
100 µg/ml	0.36 ± 0.17a	0.73 ± 0.45ab	1.22 ± 0.27b

The data represent the mean and standard deviation of three independent samples. Different lower case letters displayed on the right side of the data in each column are significantly different between the cottonseed extract concentrations at p < 0.05. Values with statistical significance in gene expression are underlined.

### Effect of cottonseed extracts on ZFP36L3 gene expression

ZFP36L3 mRNA levels were modestly affected by the coat and kernel extracts from glanded and glandless cottonseed in mouse RAW macrophages (Table 3). ZFP36L3 mRNA levels were only significantly increased to 2-fold by the kernel extract from glanded cottonseed after 8 h treatment (Table 3). qPCR did not show any significant effect of the coat and kernel extracts from glandless cottonseed on ZFP36L3 mRNA levels in mouse RAW macrophages (Table 3).

**Table 3:** Effect of Cottonseed Extracts on ZFP36L3 mRNA Levels.

Glanded coat	2 h	8 h	24 h
1% DMSO	1.00 ± 0.00a	1.00 ± 0.00a	1.00 ± 0.00a
5 µg/ml	2.24 ± 2.73a	1.00 ± 0.10a	0.96 ± 0.34a
10 µg/ml	1.08 ± 0.76a	0.93 ± 0.21a	0.93 ± 0.10a
20 µg/ml	0.40 ± 0.41a	0.64 ± 0.43a	0.93 ± 0.46a
30 µg/ml	0.34 ± 0.13a	3.49 ± 2.63a	1.23 ± 0.24a
40 µg/ml	1.45 ± 0.00a	2.84 ± 3.53a	1.38 ± 0.40a
50 µg/ml	0.30 ± 0.13a	1.51 ± 0.44b	1.80 ± 0.47b
100 µg/ml	1.15 ± 0.34a	0.79 ± 0.74a	1.51 ± 0.54a
**Glanded kernel**	**2 h**	**8 h**	**24 h**
1% DMSO	1.00 ± 0.00a	1.00 ± 0.00a	1.00 ± 0.00a
5 µg/ml	0.61 ± 0.12a	0.44 ± 0.37a	3.00 ± 0.90b
10 µg/ml	0.40 ± 0.51a	0.27 ± 0.09a	1.55 ± 0.93a
20 µg/ml	0.70 ± 0.57a	0.78 ± 0.49a	1.01 ± 0.29a
30 µg/ml	1.08 ± 0.93a	0.33 ± 0.09a	1.05 ± 0.90a
40 µg/ml	0.56 ± 0.42a	0.43 ± 0.19a	2.63 ± 1.40a
50 µg/ml	0.55 ± 0.46a	2.07 ± 0.50a	1.44 ± 0.76a
100 µg/ml	0.19 ± 0.13a	0.50 ± 0.13a	2.07 ± 0.79b
**Glandless coat**	**2 h**	**8 h**	**24 h**
1% DMSO	1.00 ± 0.00a	1.00 ± 0.00a	1.00 ± 0.00a
5 µg/ml	1.16 ± 0.33a	2.80 ± 1.23a	2.03 ± 1.59a
10 µg/ml	0.66 ± 0.24a	1.24 ± 0.87a	1.24 ± 0.42a
20 µg/ml	3.55 ± 4.54a	0.86 ± 0.48a	1.17 ± 0.10a
30 µg/ml	2.71 ± 2.85a	4.54 ± 2.96a	2.84 ± 0.32a
40 µg/ml	0.86 ± 0.30a	1.16 ± 1.30a	2.44 ± 0.60a
50 µg/ml	8.24 ± 10.56a	1.73 ± 0.53a	1.43 ± 0.73a
100 µg/ml	1.41 ± 0.82a	4.00 ± 1.63a	1.37 ± 0.46a
**Glandless kernel**	**2 h**	**8 h**	**24 h**
1% DMSO	1.00 ± 0.00a	1.00 ± 0.00a	1.00 ± 0.00a
5 µg/ml	0.32 ± 0.06a	0.42 ± 0.25a	0.86 ± 0.36a
10 µg/ml	0.28 ± 0.13a	0.53 ± 0.14b	0.77 ± 0.08b
20 µg/ml	1.28 ± 1.49a	0.40 ± 0.13a	1.86 ± 1.40a
30 µg/ml	0.36 ± 0.08a	0.43 ± 0.19a	0.92 ± 0.49a
40 µg/ml	0.22 ± 0.12a	0.88 ± 0.53a	0.97 ± 0.06a
50 µg/ml	0.39 ± 0.09a	0.40 ± 0.25a	1.86 ± 0.81b
100 µg/ml	0.28 ± 0.08a	0.77 ± 0.36ab	1.49 ± 0.65b

The data represent the mean and standard deviation of three independent samples. Different lower case letters displayed on the right side of the data in each column are significantly different between the cottonseed extract concentrations at p < 0.05. Values with statistical significance in gene expression are underlined.

## Discussion

We explored the potential of using cottonseed extracts as a safe source of plant polyphenols so that cottonseed value could be increased because plant polyphenols have been used for the prevention and treatment of various diseases. We recently showed that cottonseed extracts regulate DGAT and HuR gene expression in mouse macrophages^21,22^. In this study, we examined the effects of cottonseed extracts on the viability and mRNA levels of anti-inflammatory TTP family genes in mouse cells.

The major finding is that cottonseed extracts are harmless towards the cultured macrophages and adipocytes. As shown by MTT assays, the extracts from the coat and kernel of glanded and glandless cottonseed did not have any significant effect on cell viability after treatment for 2–24 h with up to 100 µg/mL of the extracts in culture medium. We recently developed a protocol for isolating bioactive extracts from seed coat and kernel of glanded and glandless cottonseed^20^. These cottonseed extracts are essentially free of the toxic compound gossypol with only 0.82, 0.03, 0.37 and 0 ng of gossypol per mg of the extracts from glanded coat, glanded kernel, glandless coat and glandless kernel, respectively^20^. These results suggest that cottonseed extracts are probably safe for consumption.

Another major finding is that gossypol and LPS exhibited significant inhibition on the viability of RAW macrophage but not adipocytes; the higher the concentration or the longer the treatment resulted in more severe reduction of mitochondrial activity. Our results that gossypol and LPS inhibited RAW cell viability are in contrast to the conclusion from a previous study^35^. It is unclear why the effect of gossypol and LPS on RAW cell viability in the two studies is different. One possible reason is that their study used a much higher cell density (4 × 10^5^ cells/mL) in 96-well plate and ours used ¼ of their cells in 24-well plate. Another potential reason is that the final concentrations of gossypol used in their studies were probably lower than those used in our study; but it is difficult to know since they did not report the total volume of the culture medium in their study^35^. The cytotoxic compound gossypol is known to be accumulated in the glanded cottonseed which causes male infertility^36^ but minimally present in glandless cottonseed^37^. Our results suggest that gossypol may affect immunity when over-consumption and accumulation of this toxic compound in the body.

The most significant finding was that only coat extract from glandless cottonseed significantly increased anti-inflammatory TTP mRNA levels up to 7-fold after 24 h treatment, resulted in increased TTP protein levels. This provides evidence for the potential health benefits of cottonseed extracts because plant extracts such as green tea and cinnamon extracts that can induce TTP gene expression have nutritional and therapeutic value for the prevention and/or treatment of inflammation-related diseases^11,14^. Other cottonseed extracts from the coat and kernel of glanded cottonseed and kernel of glandless cottonseed only had modestly effects on TTP mRNA levels in the macrophages. All four cottonseed extracts exhibited minor effects on the mRNA levels of ZFP36L1, ZFP36L2, and ZFP36L3 genes in the macrophage. It was shown previously that the anti-depressive compound quercetin was only identified in the glandless seed^20^. We showed here that TTP was only significantly induced by glandless cottonseed coat extract. It would be interested to test if quercetin induces TTP gene expression in future experiments.

The effect of ethanol extracts from glandless cottonseed on stimulating TTP gene expression up to 7-fold is similar to those of insulin (7 fold with 100 nM for 30 min in mouse adipocytes)^38^, cinnamon extract (2 fold with 100 µg/mL for 2 h in macrophages and 10 fold for 1.5 h in adipocytes)^13,14,16^ and green tea extract (50–140% in rat liver and muscle)^11^. Since anti-inflammatory TTP family proteins target TNF and a few other key cytokine mRNAs for their destruction by binding to the unstable 3′ AU-rich sequence^34,39–42^, these results suggest that cottonseed extract from glandless seed may have potential health benefits for inflammatory diseases. However, the fact that no effect of the cottonseed extract on the viability of macrophages or adipocytes require further in-depth studies of its inflammatory effects. It also requires additional studies to elucidate the molecular mechanism of cottonseed extract in potential regulation of inflammation via anti-inflammatory TTP action.

In conclusion, this study demonstrated that no cytotoxicity effect was observed in mouse cells treated with cottonseed extracts which are essentially gossypol-free, suggesting that cottonseed extracts are probably safe to use. We also showed that the extract from glandless cottonseed coat significantly increased anti-inflammatory TTP gene expression in macrophages with a magnitude similar to cinnamon polyphenol extract, green tea extract and insulin, suggesting health and nutritional benefits for inflammation-related diseases. We propose that glandless cottonseed can be a safe source of bioactive polyphenols with anti-inflammatory property.

## Materials and Methods

### Cottonseed and cell line

Glanded cottonseed and glandless cottonseed were provided by Richard Byler and Thomas Wedegaertner, respectively^43^. Mouse RAW264.7 macrophages and 3T3-L1 preadipocytes were purchased from American Type Culture Collection (Manassas, VA).

### Chemicals, reagents and equipment

Primer Express software (Thermo Fisher) was used to design PCR primers which were synthesized by Biosearch Technologies (Petaluma, CA). The gene names, GenBank accession numbers, amplicon sizes, and the sequences (5′ to 3′) of the forward primers and reverse primers, respectively, are described previously^38^. Chemicals were from Sigma. Cell culture reagents were from Gibco BRL (Thermo Fisher). qPCR reagents were from Bio-Rad.

### Cottonseed extracts

The ethanol extract from seed kernel was isolated by fractionation, defatting, and ethanol extraction, whereas the ethanol extract from seed coat was isolated by fractionation, defatting, acetic acid extraction, and ethanol extraction^20^. Briefly, cottonseed coat and kernel were homogenized by grinding. The kernel fraction was defatted with chloroform and hexane before ethanol extraction. The coat fraction was treated with acetic acid followed by autoclave and centrifugation before ethanol extraction. The ethanol extracts were dried to remove acetic acid and ethanol and reconstituted at 100 mg/mL in 100% DMSO. These cottonseed extracts were determined by HPLC-MS analysis to be essentially free of toxic gossypol with only 0.82 (glanded seed coat), 0.03 (glanded seed kernel), 0.37 (glandless seed coat) and 0 ng (glandless seed kernel) of gossypol per mg of the extracts^20^.

### Macrophage culture and treatment

Mouse RAW264.7 cells were maintained in DMEM+ medium containing DMEM, fetal bovine serum (10%, v/v), penicillin (100 U/mL), streptomycin (100 μg/mL), and L-glutamine (2 mM) in a water jacket CO_2_ incubator at 37 °C with 5% CO_2_ as described^34^. RAW macrophages were detached from the flask with a cell scraper and counted with a TC20 Automatic Cell Counter after stained with trypsin blue dye. For MTT and qPCR assays, the cells (0.5 mL, 1 × 10^5^ cells/mL) were subcultured in 24-well cell culture plate. Raw macrophages were treated with cottonseed extracts, gossypol, LPS and 1% DMSO as the control. For immunoblotting, RAW macrophages were cultured in 75-mL culture flask with 20 mL DMEM. The cells were treated with cottonseed extracts for 24 h before preparing extracts as described below.

### Adipocyte culture and treatment

Mouse 3T3-L1 preadipocytes were cultured at 37 °C in DMEM+ as described in macrophage culture. Preadipocytes were differentiated into adipocytes with a medium containing DMEM+, recombinant human insulin (1 μg/mL), dexamethasone (0.25 μM), and 1-isobutyl-3-methylxanthine (250 μM)^13^. After 48 h incubation, the medium was replaced with DMEM+ containing only of insulin (1 μg/mL). The medium was replaced with DMEM+ after incubation for additional 48 h, and the cells were grown for another 4–6 days. The cells were starved in DMEM without any supplementation for 4 h before being treated with cottonseed extracts and DMSO (the vehicle control, 1%).

### Cell cytotoxicity assay

Cell cytotoxicity was evaluated with MTT method with the *In Vitro* Toxicology Assay Kit essentially as described previously^20^. Cells in 500 µL medium were treated with cottonseed extracts, gossypol and LPS and incubated at 37 °C, 5% CO_2_ for 2, 5, 24 and 72 h. MTT assay reagent (50 µL thiazolyl blue tetrazolium bromide) was added to the medium, and incubated at 37 °C, 5% CO_2_ for 2 h before adding 500 µL MTT solubilization solution. The color density at A570 nm was measured by microplate spectrophotometer (Epoch) and SmartSpec plus Spectrophotometer (BioRad).

### Cell extracts, protein determination, sds-page and immunoblotting

Cell extracts were prepared according to a previously described procedure^34^. Briefly, RAW macrophages were scraped into 0.9% NaCl, transferred into falcon tube and centrifuged at 1,000 *g* for 5 min. Cells were lysed in a lysis buffer containing NaH_2_PO_4_ (50 mM), pH 7.6, NaCl (250 mM), Nonidet P-40 (0.5%), phenylmethylsulfonyl fluoride (1 mM) and protease inhibitor cocktail (Sigma, cat #P8340,1:100 dilution). The cell lysate was centrifuged at 10,000 *g* for 10 min at 4 °C. Protein concentrations in the supernatant were estimated with the Bradford method using the Protein Assay Dye Reagent Concentrate (Bio-Rad Laboratories) after the extracts were denatured with 0.5 M NaOH^44^. Polypeptides (100 µg of protein per lane) were separated by 4–20% SDS-PAGE (Life Technologies) following the standard protocol. Proteins were transferred onto nitrocellulose membrane in transfer buffer containing 0.1% SDS with iBlot Gel Transfer System (Invitrogen). The membrane was blocked with nonfat dry milk in TTBS buffer (5%) and incubated with anti-MBP-mTTP antibodies (1:5,000 dilution in the blocking buffer for 18 h) produced against recombinant MBP-mTTP in rabbits^34^. Alternatively, immunoblotting was performed using rabbit polyclonal antibodies against synthetic peptides derived from human TTP (Abcam product number ab83579, 1:500 dilution in blocking buffer). After washed with TTBS buffer, the membrane was incubated with second antibodies (goat anti-rabbit IgG (H + L) horseradish peroxidase conjugate, Bio-Rad, 1:5,000 dilution in TTBS buffer, 2–4 h). Following washing with TTBS buffer, the membrane was incubated with Amersham ECL Prime Western Blotting Detection Reagent (GE Life sciences) for 5 min and chemiluminescent intensity was captioned by ChemiDoc Touch Image system (BioRad).

### RNA extraction, cDNA synthesis and real-time qPCR analysis

RNAs were isolated from macrophages using TRIzol reagent according to a previous procedure^14^. The total RNAs were used to synthesize cDNAs by a DNA Engine Gradient Cycler essentially as described^14^. SYBR Green qPCR assays were identical to those described^45^. The reaction mixture contained total RNA-derived cDNAs (5 ng), forward primer and reverse primer (200 nM each), and 1× iQ SYBR Green Supermix and performed with CFX96 real-time system-C1000 Thermal Cycler. The thermal cycle consisted of 3 min at 95 °C, 40–50 cycles at 95 °C for 10 s, 65 °C for 30 s, and 72 °C for 30 s.

### qPCR Data analysis

The fold change in gene expression was estimated by the ΔΔ*C*_*T*_ method^16,46^. The cycle of threshold (*C*_*T*_) was obtained from 3–6 independent samples. The first delta *C*_*T*_ value (Δ*C*_*T*_) equals to the *C*_*T*_ value of the target mRNA mius the *C*_*T*_ value of the internal reference control (mouse 60 S ribosome protein 32, Rpl32) (Δ*C*_*T*_ = *C*_*T*Target_ − *C*_*T*ref_). The second delta *C*_*T*_ value (ΔΔ*C*_*T*_) equals to the Δ*C*_*T*_ of the target mRNA minus the Δ*C*_*T*_ of the calibrator (DMSO control) (ΔΔ*C*_*T*_ = Δ*C*_*T*Target_ − Δ*C*_*T*cal_). The fold change in expression equals to 2^−ΔΔ*CT*^.

### Statistics

The data represent the mean and standard deviation (n = 3–6). Statistics was conducted with ANOVA with SigmaStat 3.1 software (Systat Software). Multiple comparisons among the treatments were performed with Student-Newman-Keuls Method^16,21^. Significant difference between the treatment concentrations at p < 0.05 are indicated by the different lower case letters above each of the treatment time on the figures or in the table columns.

## Supplementary information

Supplementary Figure

## References

[1] Dowd MK, Pelitire SM, Delhom CD (2018). Seed-fiber ratio, seed index, and seed tissue and compositional propertoes of current cotton cultivars. J. Cotton Sci..

[2] Dowd MK, Pelitire SM (2006). Isolation of 6-methoxy gossypol and 6,6′-dimethoxy gossypol from Gossypium barbadense Sea Island cotton. J. Agric. Food Chem..

[3] Wang X, Howell CP, Chen F, Yin J, Jiang Y (2009). Gossypol–a polyphenolic compound from cotton plant. Adv. Food Nutr. Res..

[4] Kenar JA (2006). Reaction chemistry of gossypol and its derivatives. J. Am. Oil Chem. Soc..

[5] Piccinelli AL, Veneziano A, Passi S, Simone FD, Rastrelli L (2007). Flavonol glycosides from whole cottonseed by-product. Food Chem..

[6] Gao D, Cao Y, Li H (2010). Antioxidant activity of peptide fractions derived from cottonseed protein hydrolysate. J. Sci. Food Agric..

[7] Zhang QJ, Yang M, Zhao YM, Luan XH, Ke YG (2001). Isolation and structure identification of flavonol glycosides from glandless cotton seeds. Acta Pharmaceutica Sin..

[8] Dixon RA, Xie DY, Sharma SB (2005). Proanthocyanidins–a final frontier in flavonoid research?. N. Phytol..

[9] Prior RL, Gu L (2005). Occurrence and biological significance of proanthocyanidins in the American diet. Phytochemistry.

[10] Yang CS, Landau JM, Huang MT, Newmark HL (2001). Inhibition of carcinogenesis by dietary polyphenolic compounds. Annu. Rev. Nutr..

[11] Cao H (2007). Green tea increases anti-inflammatory tristetraprolin and decreases pro-inflammatory tumor necrosis factor mRNA levels in rats. J. Inflamm. (Lond.).

[12] Cao H (2007). Green tea polyphenol extract regulates the expression of genes involved in glucose uptake and insulin signaling in rats fed a high fructose diet. J. Agric. Food Chem..

[13] Cao H, Polansky MM, Anderson RA (2007). Cinnamon extract and polyphenols affect the expression of tristetraprolin, insulin receptor, and glucose transporter 4 in mouse 3T3-L1 adipocytes. Arch. Biochem. Biophys..

[14] Cao H, Urban JF, Anderson RA (2008). Cinnamon polyphenol extract affects immune responses by regulating anti- and proinflammatory and glucose transporter gene expression in mouse macrophages. J. Nutr..

[15] Cao H, Graves DJ, Anderson RA (2010). Cinnamon extract regulates glucose transporter and insulin-signaling gene expression in mouse adipocytes. Phytomedicine..

[16] Cao H, Anderson RA (2011). Cinnamon polyphenol extract regulates tristetraprolin and related gene expression in mouse adipocytes. J. Agric. Food Chem..

[17] Cao Heping, Sethumadhavan Kandan, Li Ke, Boue Stephen M., Anderson Richard A. (2019). Cinnamon Polyphenol Extract and Insulin Regulate Diacylglycerol Acyltransferase Gene Expression in Mouse Adipocytes and Macrophages. Plant Foods for Human Nutrition.

[18] Anderson RA, Polansky MM (2002). Tea enhances insulin activity. J. Agric. Food Chem..

[19] Anderson RA (2004). Isolation and characterization of polyphenol type-A polymers from cinnamon with insulin-like biological activity. J. Agric. Food Chem..

[20] Cao H, Sethumadhavan K, Bland JM (2018). Isolation of Cottonseed Extracts That Affect Human Cancer Cell Growth. Sci. Rep..

[21] Cao H, Sethumadhavan K (2018). Cottonseed Extracts and Gossypol Regulate Diacylglycerol Acyltransferase Gene Expression in Mouse Macrophages. J. Agric. Food Chem..

[22] Cao H, Sethumadhavan K (2019). Gossypol but not cottonseed extracts or lipopolysaccharides stimulates HuR gene expression in mouse cells. J. Funct. Foods.

[23] Fu M, Blackshear PJ (2017). RNA-binding proteins in immune regulation: a focus on CCCH zinc finger proteins. Nat. Rev. Immunol..

[24] Patial S, Blackshear PJ (2016). Tristetraprolin as a Therapeutic Target in Inflammatory Disease. Trends Pharmacol. Sci..

[25] Blackshear PJ (2002). Tristetraprolin and other CCCH tandem zinc-finger proteins in the regulation of mRNA turnover. Biochem. Soc. Trans..

[26] Blackshear PJ (2005). Zfp36l3, a rodent X chromosome gene encoding a placenta-specific member of the Tristetraprolin family of CCCH tandem zinc finger proteins. Biol. Reprod..

[27] Cao H, Deterding LJ, Blackshear PJ (2007). Phosphorylation site analysis of the anti-inflammatory and mRNA-destabilizing protein tristetraprolin [Review]. Expert. Rev. Proteom..

[28] Cao H, Deterding LJ, Blackshear PJ (2014). Identification of a major phosphopeptide in human tristetraprolin by phosphopeptide mapping and mass spectrometry. PLoS ONE.

[29] Phillips K, Kedersha N, Shen L, Blackshear PJ, Anderson P (2004). Arthritis suppressor genes TIA-1 and TTP dampen the expression of tumor necrosis factor alpha, cyclooxygenase 2, and inflammatory arthritis. Proc. Natl. Acad. Sci. USA.

[30] Taylor GA (1996). A pathogenetic role for TNF alpha in the syndrome of cachexia, arthritis, and autoimmunity resulting from tristetraprolin (TTP) deficiency. Immunity.

[31] Sauer I (2006). Interferons limit inflammatory responses by induction of tristetraprolin. Blood.

[32] Astakhova AA, Chistyakov DV, Sergeeva MG, Reiser G (2018). Regulation of the ARE-binding proteins, TTP (tristetraprolin) and HuR (human antigen R), in inflammatory response in astrocytes. Neurochem. Int..

[33] Mazan-Mamczarz K (2003). RNA-binding protein HuR enhances p53 translation in response to ultraviolet light irradiation. Proc. Natl. Acad. Sci. USA.

[34] Cao H, Tuttle JS, Blackshear PJ (2004). Immunological characterization of tristetraprolin as a low abundance, inducible, stable cytosolic protein. J. Biol. Chem..

[35] Huo M (2013). Suppression of LPS-induced inflammatory responses by gossypol in RAW 264.7 cells and mouse models. Int. Immunopharmacol..

[36] Coutinho EM (2002). Gossypol: a contraceptive for men. Contraception.

[37] Lusas EW, Jividen GM (1987). Glandless cottonseed: a review of the first 25 years of processing and utilization research. J. Am. Oil Chem. Soc..

[38] Cao H, Urban JF, Anderson RA (2008). Insulin increases tristetraprolin and decreases VEGF gene expression in mouse 3T3-L1 adipocytes. Obes. (Silver. Spring).

[39] Cao H (2004). Expression, purification, and biochemical characterization of the antiinflammatory tristetraprolin: a zinc-dependent mRNA binding protein affected by posttranslational modifications. Biochemistry.

[40] Cao H (2006). Identification of the anti-inflammatory protein tristetraprolin as a hyperphosphorylated protein by mass spectrometry and site-directed mutagenesis. Biochem. J..

[41] Carballo E, Lai WS, Blackshear PJ (1998). Feedback inhibition of macrophage tumor necrosis factor-alpha production by tristetraprolin. Science.

[42] Lai WS (1999). Evidence that tristetraprolin binds to AU-rich elements and promotes the deadenylation and destabilization of tumor necrosis factor alpha mRNA. Mol. Cell. Biol..

[43] Zhang J (2016). Registration of ‘NuMex COT 15 GLS’ glandless cotton. J. Plant. Registrations.

[44] Cao H, Sullivan TD, Boyer CD, Shannon JC (1995). *Bt1*, a structural gene for the major 39-44 kDa amyloplast membrane polypeptides. Physiol. Plant..

[45] Cao H, Shockey JM (2012). Comparison of TaqMan and SYBR Green qPCR methods for quantitative gene expression in tung tree tissues. J. Agric. Food Chem..

[46] Livak KJ, Schmittgen TD (2001). Analysis of relative gene expression data using real-time quantitative PCR and the 2(-Delta Delta C(T)) Method. Methods.

