# Supplementary material for: Regulation of Cell Viability and Anti-inflammatory Tristetraprolin Family Gene Expression in Mouse Macrophages by Cottonseed Extracts
Source: Sci Rep. 2020 Jan 21;10:775. doi: 10.1038/s41598-020-57584-9 (PMC6972847; doi:10.1038/s41598-020-57584-9)
Supplement: Supplementary file 1 — Supplementary Figure [file 41598_2020_57584_MOESM1_ESM.pdf]

# Regulation of Cell Viability and Anti-inflammatory Tristetraprolin Family Gene Expression in Mouse Macrophages by Cottonseed Extracts

**Heping Cao & Kandan Sethumadhavan**
United States Department of Agriculture, Agricultural Research Service, Southern Regional Research Center, 1100 Robert E. Lee Boulevard, New Orleans, LA, 70124, USA. Correspondence and requests for materials should be addressed to H.C. (email: Heping.Cao@ars.usda.gov). **ORCID (**Heping Cao: 0000-0002-0958-1468)

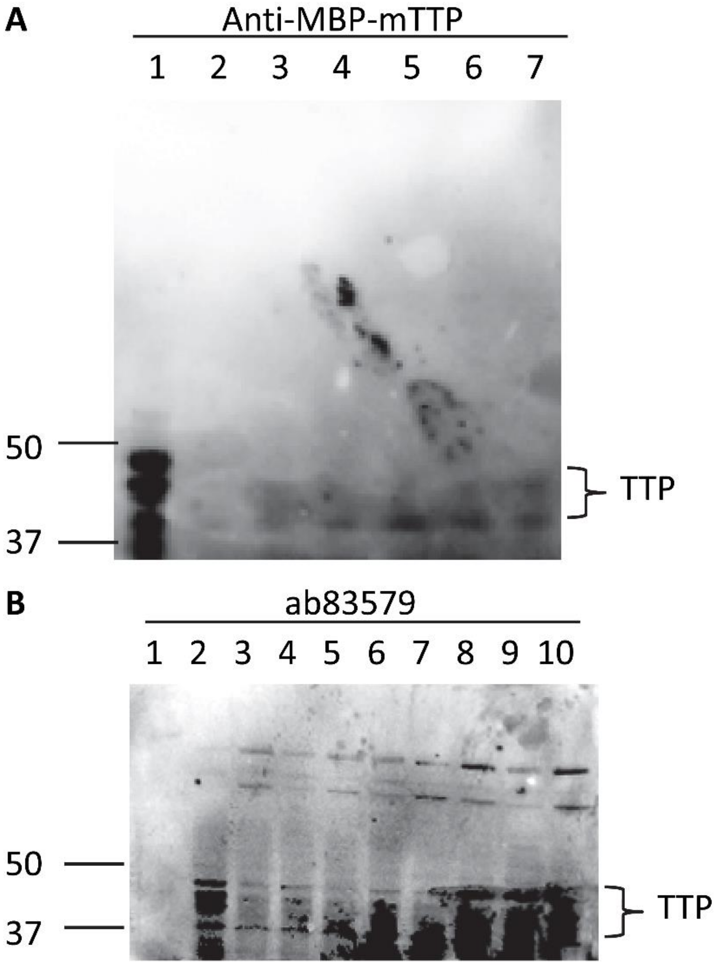


**Supplementary Figure.** Effect of LPS and cottonseed extracts on TTP protein expression. Mouse RAW264.7 cells were stimulated with LPS and cottonseed extracts for various times. Cell extract was used for immunoblotting with the anti-MBP-mTTP serum or synthetic peptide ab83579 polyclonal antibodies. The blot was incubated in the primary antibodies for 18 h and the secondary antibody for 4 h. (A) anti-MBP-mTTP serum, lane 1: LPS (2 h, 100 ng/mL), lanes 2-7: glandless cottonseed coat extract (24 h, 5, 10, 20, 30, 40 and 100 µg/mL, respectively). (B) ab83579, lane 1: protein standards, lane 2: LPS (2 h, 100 ng/mL), lanes 3-6: glanded cottonseed coat extract (100 µg/mL, 2, 4, 8 and 24 h, respectively), lanes 7-10: glandless cottonseed coat extract (100 µg/mL, 2, 4, 8 and 24 h, respectively).
